# Adult-Onset Still’s Disease (AOSD) Associated With Collagenous Colitis (CC) and Basedow’s Disease: A Case Report

**DOI:** 10.7759/cureus.42192

**Published:** 2023-07-20

**Authors:** Alina Stiuliuc, Abeline Kapuczinski, Marjolaine Weynand, Camille Lemoine, Muhammad Soyfoo

**Affiliations:** 1 Rheumatology, Erasmus Hospital, Brussels, BEL; 2 Rheumatology, CHU Brugmann, Université Libre de Bruxelles, Brussels, BEL

**Keywords:** thyroid orbitopathy, inflammatory disease, collagenous colitis, basedow’s disease, adult onset still's disease (aosd)

## Abstract

Adult-onset Still’s disease (AOSD) is a rare auto-inflammatory syndrome of unknown etiology. Basedow’s disease is a common cause of auto-immune hyperthyroidism. Collagenous colitis (CC) is a form of microscopic colitis (MC) affecting predominantly young women. While the etiology of the disease remains unclear, some studies suggest the role of auto-immunity. The association between AOSD and Basedow’s disease has been reported in previous cases, suggesting auto-inflammation as a potential trigger of relapsing thyroid dysfunction. Although the co-existence of AOSD with inflammatory gastrointestinal disorders such as Crohn’s disease and ulcerative colitis has also been described, we did not find any correlation with MC in the literature. We here describe the case of a woman having AOSD associated with Basedow’s disease and CC.

## Introduction

Adult-onset Still’s disease (AOSD) is a rare auto-inflammatory syndrome of unknown etiology with an estimated incidence of 0.16 and 0.62 per 100,000 individuals worldwide. Its clinical spectrum is pleiotropic, more consistent with spiking fever, arthritis, pruritic or nonpruritic evanescent rash, pharyngitis, and lymphadenopathy, while polyserositis and hepatitis are less frequent [1-3].

While there are no currently specific biological markers, the laboratory test shows high levels of erythrocyte sedimentation rate and C-reactive protein (CRP); neutrophil leucocytosis and high ferritin levels characterized by a decreased glycosylated ferritin (<20%) [1, 3].

The diagnosis is based on the combination of clinical and laboratory findings as well as the exclusion of other autoimmune, inflammatory, infectious, or neoplastic disorders. Yamaguchi’s criteria are the most widely used due to higher sensitivity (96.2%) and specificity (92.1%). The disease evolution and severity are heterogeneous. Macrophage activation syndrome (MAS) is the most severe complication with a high mortality rate [4-5].

Basedow's disease is a common cause of auto-immune hyperthyroidism in which thyroid-stimulating antibodies activate thyroid-stimulating hormone (TSH) receptors, thus inducing thyroid hormone synthesis. Pathognomonic features include ophthalmopathy and pre-tibial edema while other clinical signs are namely fatigue, weight loss, tachycardia, sleep disturbances, hand tremors, frequent bowel movements or diarrhea, and difficulty tolerating hot conditions reflecting accelerated metabolism [6-7].

Collagenous colitis (CC) is a form of microscopic colitis (MC) affecting predominantly young women, with a pooled incidence rate of 4.14 per 100,000 person-years. The hallmark of the disease is watery diarrhea with normal endoscopic findings; other symptoms include fatigue, weight loss, abdominal pain, and urgency fecal incontinence. While the etiology of the disease remains unclear, some studies suggest the role of auto-immunity [8-12].

 The association between AOSD and Basedow’s disease has been reported in previous cases, suggesting auto-inflammation as a potential trigger of relapsing thyroid dysfunction [13-14]. Although the co-existence of AOSD with inflammatory gastrointestinal disorders such as Crohn's disease [15] and ulcerative colitis [16] has also been described, we did not find any correlation with MC.

 We here describe the case of a Portuguese woman having Basedow's disease associated with AOSD and CC.

## Case presentation

A 36-year-old woman of Portuguese origin came to the Internal Medicine Department outpatient clinic because of a rash resembling urticaria on her right leg. From her medical history, we note that she was diagnosed with Basedow's disease and left orbitopathy some years prior in Portugal and she was treated with Strumazol (thiamazole) 5 mg every two days.

The skin lesions started in January 2022 in Portugal three weeks after the SARS-CoV-2 infection despite complete vaccination. The rash was daily continuing until May 2022 looking like maculopapular lesions and even almost purpuric lesions affecting all four limbs, sometimes her trunk. Rarely, the rash may appear on her face like a slight malar rash. She also reported a fever since January 2022 fluctuating between 39.0°C and 40°C and recurrent sore throat. In early February, the patient developed permanent arthralgia with an inflammatory appearance, especially on both wrists, metacarpophalangeal, and proximal interphalangeal joints with macular lesions in relation to arthralgia. NSAIDs (non-steroidal anti-inflammatory drugs) were prescribed to the patient during a consultation in Portugal (Naproxen 500 mg) with the improvement of the fever for a short period before relapsing. Prednisolone 60 mg daily was introduced with tapering doses until 15 mg/day.

**Figure 1:**
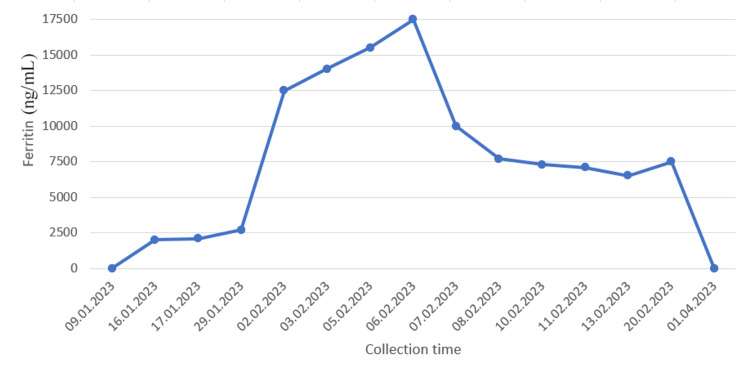
Ferritin levels over time.

The patient was hospitalized in Belgium in September 2022 in Hematology Department because of suspicion of lymphoma. Blood tests showed biological inflammatory syndrome with CRP of 40 mg/L (normal value <5 mg/L), anemia, lactate dehydrogenase (LDH) 800 UI/L (normal value 135-214 UI/L) without schistocytes, and high haptoglobin and ferritin with increasing values (Figure 1).

Protein electrophoresis was normal. GOT (glutamic-oxal acetic transaminase) was raised (70 U/L, normal value <32). The complement level was normal, RF (rheumatoid factor) was slightly positive (46 UI/mL), antinuclear antibodies, and anti-cyclic citrullinated peptide were negative. All infectious serologies were tested negative: HBV (hepatitis B virus), HCV (hepatitis C virus), HIV (human immunodeficiency virus), syphilis, coxiella, rickettsia conori and mooseri, Borrelia, malaria, and leishmania. Serological tests for EBV (Epstein-Barr virus), CMV (cytomegalovirus), and toxoplasma were positive indicating an old infection. Blood cultures were also negative. 

The thyroid labs showed normal thyroid stimulating hormone (TSH) and FT4 levels. We debated the potential role of Strumazol in some of the features the patient displayed: fever, rash, arthralgia, and high transaminase levels; the treatment was discontinued with no clinical or biological improvement and was later resumed under the endocrinologist's advice.

**Figure 2:**
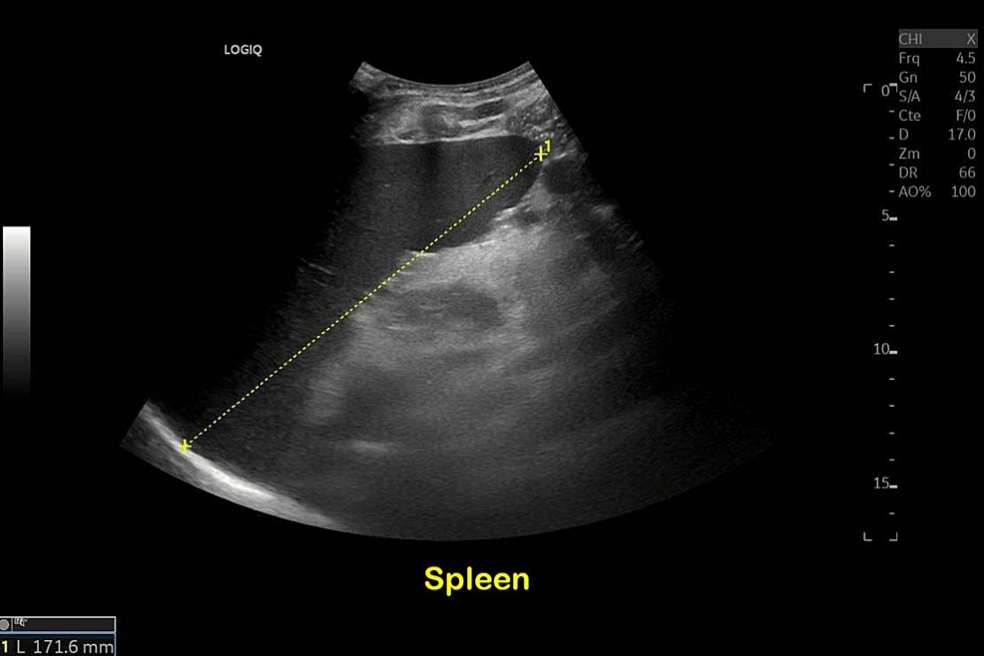
Splenomegaly on abdominal echography.

Further investigation by PET-CT scan showed hypermetabolic splenomegaly and hepatomegaly with supradiaphragmatic lymph nodes. Thoraco-abdominal CT scan and an abdominal ultrasound confirmed the hepatosplenomegaly (Figure 2) but also showed a diffuse infiltration of the mesenteric fat, a pelvic intraperitoneal effusion, and a small pericardial and pleural effusion. Bone marrow biopsy was negative as well as abdominal fat biopsy and lymph nodes biopsy did not reveal any signs of malignancy. The cardiac ultrasound was also normal.

In the meantime, the patient also complained of disabling diarrhea. Repeated stool cultures were negative; the screening for celiac disease was also negative. A colonoscopy assessment was done earlier in September 2022, displaying inflammation in lamina propria and focal thick collagenous subepithelial bands in favor of CC. Moreover, the symptoms improved with corticoids.

Given the absence of infectious or neoplastic findings, AOSD diagnosis is discussed based on the association of fever, consistent skin lesions, sore throat, biological inflammatory syndrome, elevated ferritin and transaminases, arthralgia, reactive lymphadenopathies, and hepatosplenomegaly. Macrophage activation syndrome was excluded.

In November 2022, the patient was referred to our Rheumatology Department. She took hydrocortisone 10 mg at that time. Ultrasound staging of both her hands and wrists showed a left wrist effusion with grayscale grade 2 without a power-Doppler signal and a right wrist synovitis with grayscale grade 2 and power-Doppler signal grade 1.

Treatment with ledertrexate 10 mg per week with folic acid complement per day was initiated. Despite this, the clinical and biological picture did not improve. In December 2022, subcutaneous tocilizumab 162 mg once a week was added to ledertrexate without efficiency. Treatment was switched to Anakinra 100 mg once a day in January 2023 with clinical improvement of fever and rash but was stopped because of a hypersensitivity reaction. Finally, tocilizumab IV and methylprednisolone 32 mg/day were initiated in February 2023 with a good clinical and biological response. The patient had a regular follow-up in our Endocrinology Department. In January 2023 the thyroid labs showed normal TSH and FT4, thyroid stimulating hormone receptor antibodies (TRAb) levels of 0.50 U/L with anti-TPO (antithyroperoxidase antibodies) at 179 kUI/L. 

Further investigations showed echographic images compatible with thyroiditis without nodules and diffuse scintigraphy hypocaptation. Strumazol was finally discontinued. Thyroid labs were later performed indicating hypothyroidism (TSH 7 mU/L in February 2023). Treatment with L-thyroxine 25/50 µg per day was initiated.

**Figure 3:**
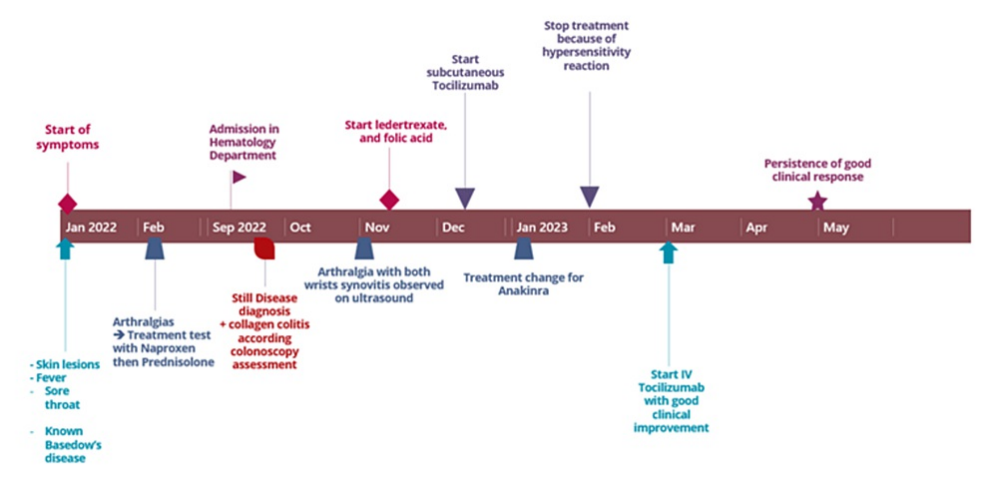
Summary of the patient's evolution over time.

The last follow-up in our hospital was in April 2023. The patient only complained of mild carpal arthralgia, responding to 8 mg of methylprednisolone per day; no other systemic signs or symptoms were reported. The ferritin levels, liver enzymes, and CRP were in the normal range. The thyroid labs showed normal values of TSH and FT4, TRAb titers, and anti-TPO antibodies of 0.31 U/L and 199 kUI/L, respectively. Treatment with L-thyroxine 25/50 µg/day was maintained and a follow-up was proposed in 3 months. Figure 3 shows the summary of the patient’s clinical history.

## Discussion

We present here the case of a Portuguese patient concomitant Basedow's disease, AOSD, and CC. As far as we know, the association between the three disorders has never been reported.

First described in 1971 by Bywaters on a series of 14 patients displaying features resembling chronic juvenile arthritis [17], AOSD is a rare systemic disorder at the crossroad of auto-inflammatory and auto-immune diseases, with a complex physiopathology involving both innate and adaptive immunity. The clinical presentation may thus mimic other auto-inflammatory or auto-immune disorders, infection, and malignant diseases, manifesting typically with spiking fever, evanescent rash, arthralgia, pharyngitis, lymphadenopathy, hepato-splenomegaly and in some cases hepatitis of serositis [1-2].

The diagnosis relies on clinical manifestations correlated with biological markers (neutrophil leucocytosis, hyperferritinemia, high inflammatory markers, after the exclusion of the mimic disorders). Yamaguchi criteria are most commonly used (Table 1) [18].

**Table 1:** Yamaguchi criteria for AOSD. AOSD, adult-onset Still's disease

Major criteria	Fever ≥39°C persisting for ≥1 week
	Arthralgia/arthritis persisting for ≥2 weeks
	Typical rash
	White blood cell count ≥10×10^9^/L (>80% neutrophils)
Minor criteria	Sore throat
	Lymphadenopathy and/or splenomegaly
	Increased serum aminotransferase or lactate dehydrogenase levels (after other causes have been excluded)
	-Negative IgM rheumatoid factor and antinuclear antibodies (immunofluorescence assay)
Exclusion criteria	Infections, in particular sepsis and infectious mononucleosis
	Malignance, in particular lymphoma
	Other rheumatic diseases, in particular polyarteritis nodosa and vasculitis in the course of rheumatoid arthritis
For the diagnosis of AOSD, ≥5 criteria must be met, including ≥2 major. In patients with any of the exclusion criteria, the diagnosis is excluded. The sensitivity of the criteria is 80.6%, and the specificity is 98.5%.	

Basedow’s disease belongs to the specter of autoimmune thyroid diseases (AITD), alongside Hashimoto’s disease. It was first described in 1835 by Robert Graves and it is characterized by the presence of TRAb, leading to hyperfunction of the thyroid. The typical clinical presentation is the Merseburger triad, consisting of thyreotocicosis, ophthalmopathy, and diffuse goiter; dermopathy (pre-tibial edema) is also considered patognomonicalAQ, although less prevalent. The diagnosis of the disease relies on clinical and laboratory findings (low concentration of TSH , high Ft4, and concentration of TRAb) [6-7].

Collagenous colitis is a type of MC, besides lymphocytic colitis (LC), first described in 1973. Typical manifestations include watery diarrhea, a hallmark of MC; other symptoms include fatigue, weight loss, abdominal pain, and fecal incontinence that might mimic irritable bowel syndrome (IBS) and cannot distinguish CC from LC. While some individuals may have painful, disabling symptoms, others only experience mild discomfort or no symptoms at all. A recent case report described by Sugiyama and colleagues describes colonic perforation as a complication of CC in a patient without diarrheal symptoms. The endoscopic evaluation of the colon is usually normal although erythema, edema, change in vascularity mucosal tears, and nodularity have been described. The diagnosis relies on histological findings in colonic mucosal biopsies such as thickened collagen bands under the surface of the epithelium [8-12].

At the presentation in our Rheumatology Department, our patient already had a diagnosis of Basedow's disease and ophthalmopathy made in Portugal and was treated with Strumazol. The thyroid hormones in the baseline were normal. The possible role of Strumazol was previously debated and the treatment was temporarily suspended with no clinical improvement and later resumed under the advice of the endocrinology team.

The diagnosis of AOSD was then established based on clinical manifestations: fever, rash, arthralgia, sore throat, lymphadenopathy, hepato-splenomegaly, and biological findings: neutrophil leucocytosis, high ferritin levels, elevated inflammatory markers, thus fulfilling all major and minor Yamaguchi criteria, except for the negativity of the rheumatoid factor. An extensive evaluation was performed previously in order to discharge other possible causes such as infection or malignancy. Concomitantly with these symptoms, the patient reported watery diarrhea.

 The infectious causes and celiac disease were excluded and a previous colonoscopy with biopsies was performed in September 2022 revealing inflammation in lamina propria and focal thick collagenous subepithelial bands. Based on these endoscopic findings, the diagnosis of CC was established. The symptoms improved under glucocorticoids. This aspect can be interpreted in various ways. NSAIDs have been incriminated in the etiology of CC and at that time, our patient was on high doses of Naproxen; this indicates that the cause of the intestinal disorder might have been iatrogenic.

From another point of view, considering the context of concomitant AODS and Basedow’s disease, the interplay of the auto-immunity cannot be excluded. We will first discuss a possible common physiopathological link between the three disorders.

The association between AODS and Basedow’s disease has been previously reported in a few case reports. Masataka et al.’s report in 2011 regarding the case of a 50-year-old woman with AOSD and relapsing Basedow’s disease, suggests their pathogenesis may be intricated [14]. In the abstract, the mentioned authors refer to other five clinical cases with the co-existence of both diseases.

A review of the literature by Hu et al. was published in 2014 regarding AOSD association with thyroid dysfunction [13]. The authors disclosed the case of a 43-year-old woman with pre-existing Basedow’s disease that was aggravated by active AOSD and referred further to a similar case reported by Chen et al. A common physiopathological link was debated and a screening for one disease in the presence of another was proposed. While the physiopathology of AOSD and Basedow’s disease is multi-factorial and complex, they share indeed some common aspects.

First of all, genetically, by the presence of HLA-DRB1 alleles. Second, by immune mechanisms; a Th1-oriented response with elevated concentrations of TNF alpha, IL-6, and IL-18 predominates the immunopathogenesis of AOSD disease. IL-18 plays a major role in the inflammatory cascade, inducing other cytokines such as IL-1beta, IL-8, TNF alpha, and INF gamma. IL-1 beta alters the expression of junction proteins in human thyrocytes and is linked to auto-immune thyroid disorders [1, 5-6, 13, 19].

High levels of CXCL10, a chemokine-induced by INF gamma were found in the serum of AOSD and Basedow patients, both compared to other auto-immune diseases and healthy controls [5, 19-21]. In conclusion, AOSD and Basedow’s disease share molecular mimicry on the IFN gamma and interleukin levels, as previously reported [13]. As stated before, we did not find any connection between MC and AOSD, but our research revealed a possible link with Basedow’s disease. In an observational study, 75 patients with CC displayed concomitantly auto-immune disorders, most frequently Hashimoto thyroiditis, rheumatoid arthritis, lupus and Sjogren syndrome, while Basedow’s disease was reported in one case [22].

In a recent systematic review of the literature conducted by Zabana et al. [12], MC with the predominance of CC was associated in 50% of cases with a series of auto-immune diseases, most commonly: celiac disease, autoimmune thyroid disease, type one diabetes, spondyloarthritis, rheumatoid arthritis, seronegative arthritis, Sjogren syndrome, lupus and scleroderma/CREST syndrome. Amongst the rarest associations, we cite SAPHO syndrome and polyarthritis.

The role of auto-immunity in the pathogenesis of CC has been debated. An aberrant T lymphocyte response may drive chronic gut inflammation with the predominance of a Th1 cytokine profile with elevated levels of INF gamma, TNF alpha, and IL-1 beta. In a series of 18 biopsies, MC revealed a TH1 mucosal cytokine profile with the INF gamma as the predominantly upregulated cytokine pattern that might suggest a response to a luminal antigen [8, 10]. It is thus possible that in our case, the three clinical entities, although not simultaneously diagnosed, are linked by the bias of auto-immunity. Another aspect to discuss is the treatment strategy.

There is no currently internationally accepted consensus regarding AOSD management and a treat-to-target strategy is absent. Due to a lack of clinically controlled trials, the treatment remains empirical, aiming to block inflammation at early stages, thus preventing persistent symptoms, organ damage, and potentially life-threatening complications such as macrophage activation syndrome. The first clinical practice guidelines were established in 2017 by the Japanese Ministry of Health. Systemic glucocorticoids with high doses of IV pulses were proposed for patients with severe organ involvement and methotrexate was suggested in cases of steroid resistance [5].

NSAIDs and corticosteroids are commonly used as first-line therapy, followed by csDMARDS as steroid-sparing agents. As mentioned previously, inflammatory cytokines play a pivotal role in the systemic inflammatory response and are essential mediators in AOSD pathophysiology, thus cytokine-blocking medicine emerged as an appropriate treatment [3, 5, 23].

The role of IL-1 inhibition was emphasized in a systematic review conducted by Kedor et al. [23]. The current biological agents are Anakinra, an IL-1R antagonist, Canakinumab (an anti-IL-1 antibody), and rilonacept (a soluble IL-1 trap molecule). Tocilizumab, an IL-6 receptor, should be considered as an alternative to IL-1 antagonists, particularly when joint involvement is present [5, 23].

Other therapeutical options are emerging; in a multicentric phase II clinical trial conducted on 23 patients with AOSD, tadekining alpha (IL-18BP), the recombinant human IL-18 binding protein, showed a favorable safety profile and was associated with early signs of efficacy [24]. Our patient was first treated in Portugal with NSAIDs with a short improvement of fever, then relapse, and then corticoids were added with tapering doses. When referred to our Department, she displayed signs of active disease.

Methotrexate was added as a corticoid-sparing agent and after a month Tocilizumab 162 mg/week was added. Our first biological treatment choice was motivated by the persistent active disease of polyarthritis. Additionally, the 2021 EUGOGO (European Group on Graves' orbitopathy) clinical practice guidelines support the efficacity of Tocilizumab in the management of the disease [25]. Despite this approach, an optimal control of the disease was not attained and a switch to Anakinra was proposed, with a convenient clinical and biological response first, followed by a hypersensitivity reaction which led to its discontinuation.

Finally, Tocilizumab iv and Methylprednisolone 32 mg were added with good tolerance and clinical response. The thyroid disorder was monitored concomitantly by our endocrinology team. Strumazol was finally discontinued in January 2023 due to hypothyroidism and L-thyroxine was initiated (25/50 µg/per day). At her last follow-up in April 2023, the patient was in clinical remission, reporting only mild arthralgias with no synovitis, well responding to glucocorticoids (8 mg of methylprednisolone). The inflammatory markers, the transaminase levels, the TSH, and FT4 were in the normal range. Tocilizumab and L-thyroxine were continued; glucocorticoids were further tapered.

## Conclusions

We presented a case of coexisting hyperthyroidism, AOSD, and CC. Although not diagnosed simultaneously, the development of these disorders in the same patient encouraged us to investigate a possible common immunologic link. It is important to keep in mind that AOSD physiopathology is very complex involving both native and adaptive immunity. From this background, other immunological diseases might be triggered or revealed. Our patient was already diagnosed with Basedow’s disease and developed auto-immune hypothyroidism in the follow-up, while on biological treatment. Previous data revealed that AOSD and Basedow's disease share some common aspects like molecular mimicry on the IFN gamma and interleukin level. We did not find any link between AOSD and CC but our literature research supports the association between MC and thyroid dysfunction. Further insight into MC's physiopathology shows similarities with AOSD and auto-immune thyroid disorders. It is thus possible, that in this case, these clinical entities are linked by the bias of auto-immunity.
